# Fetid Breath

**Published:** 1871-07

**Authors:** 


*Fœtid Breath.*—If it proceeds from a carious tooth, filling or extracting the tooth will effect its instaneous disappearance; if from want of cleanliness, a few washings of the mouth and teeth with some slightly alcoholic lotion will readily remove it. If its origin is in the stomach, bicarbonate of soda, or charcoal taken internally 2 or 3 times a day, for a number of days will neutralize and cure it. When the odor is very strong, or is due to ozœna, or does not yield to the above named means, the following may be used several times a day as a wash, gargle or, by means of a spray instrument, also swallowing a tea or dessert spoonful; take of carbolic acid 15 grains, alcohol a fluid-ounce, water 33 fluid-ounces; mix.—Or else, take of permanganate of potassa 154 grains, water 33 fluid-ounces; mix.

